# Differences in microbiota profiles of simple and complicated pediatric appendicitis cases

**DOI:** 10.3389/fcimb.2026.1966569

**Published:** 2026-10-05

**Authors:** Lütfiye Öksüz, Evrim Özdemir, İdil Tunçer, Seçil Yüksel, Tayyip Karaman, Başak Erginel

**Affiliations:** 1 Department of Medical Microbiology, Istanbul Faculty of Medicine, Istanbul University, Istanbul, Türkiye; 2 Medical Laboratory Programme, Vocational School, Istanbul Atlas University, Istanbul, Türkiye; 3 Institute of Graduate Studies in Health Sciences, Istanbul University, Istanbul, Türkiye; 4 Department of Pediatric Surgery, Istanbul Faculty of Medicine, Istanbul, Istanbul University, Istanbul, Türkiye; 5 Department of Biostatistics and Medical Informatics, Faculty of Medicine, Acıbadem Mehmet Ali Aydınlar University, Istanbul, Türkiye

**Keywords:** complicated appendicitis, microbiota analysis, pre-operative treatment, rectal swab, simple appendicitis

## Abstract

This study aimed to identify the differences in microbial composition between simple appendicitis (SA) and complicated appendicitis (CA) in a limited sample size using culture and microbiota analysis. Appendix tissues and rectal swabs for microbiota analysis and conventional cultures were collected from patients. 16S rRNA regions were amplified using a DNA preparation kit with primers designed to target the V3-V4 region. Sixteen (41%) of the patients were classified as CA based on the histopathological analysis, and the rest as SA. The effect size was slightly greater in rectal swab samples than in appendiceal tissue samples. Based on the conventional culture, the most common aerobic bacteria were *Escherichia*, *Klebsiella* spp., and *Enterococcus* spp. A decrease in both Pseudomonadota and Bacteriodota phyla was observed in tissue samples, while a decrease in Bacteriodota and an increase in Pseudomonadota were observed in rectal samples. Differences in the relative abundance of *Coprobacter* and *Hungatella* were observed between CAT and SAT samples; however, no genus-level difference remained significant after ANCOM-BC2 multiple-testing correction. The most notable feature of the CAT group was the relatively higher presence of potential pathobionts. *Bacteriodes* and *Roseburia* showed higher relative abundance in the SA rectal (SAR) group. The genera *Eubacterium* and *Clostridioides* showed a significant increase in both the SAR and CA rectal (CAR) groups. Lower relative abundances of *Parvimonas, Prevotella*, and *Dialister* and a higher relative abundance of *Tyzzerella* were observed in CAR compared with controls, although these differences did not remain significant after multiple-testing correction. In the ANCOM-BC2 pairwise analysis, an unclassified genus within *Enterobacteriaceae* was significantly enriched in CAR compared with healthy controls (LFC = 3.56, q = 0.003). No statistically significant distinction was shown at the alpha and beta diversity levels between SA and CA cases. Our findings support previous studies on increased density and diversity at the phylum level in the CA group. The main limitations were the limited sample size and the use of pre-operative antibiotics. Microbiota profiling of appendiceal tissue and rectal swab samples may provide complementary information for understanding microbiota-associated patterns in appendicitis; however, the diagnostic or predictive utility of these microbial profiles requires validation in larger, independent cohorts using dedicated predictive modeling and diagnostic performance analyses.

## 1 Introduction

The appendix, a 10 cm long, worm-like sac that emerges from the junction between the small and large intestines, was for years considered a persistent tissue remnant with no real function, but it was later understood to be a modest lymphatic organ that protects the intestines from pathogens. Later studies provided strong evidence that the appendix plays an additional role in harboring and supporting the normal resident microbiota, thus enabling the colon to rapidly regenerate itself after diarrhea or other bowel disorders (Chess, 2024). Acute appendicitis (AA), caused by inflammation of the appendix, is the most common reason for emergency abdominal surgery in children (Stringer, 2017; Liu et al., 2025; Salminen et al., 2025). AA is diagnosed in 1% to 8% of children presenting to pediatric emergency departments with acute abdominal pain. AA develops due to luminal obstruction that can occur due to fecaliths, foreign bodies, malignancy, or lymphoid hyperplasia. However, it is suggested that the pathogenesis of AA may be more closely related to dysbiosis of microbiota (Liu et al., 2025). The appendix microbiota can change significantly in various diseases. In AA, a decrease in microbial diversity and an increase in potentially pathogenic bacteria such as *Parvimonas* and *Acinetobacter* are observed, which may contribute to the inflammatory characteristics (Asnicar et al., 2021; Sagor et al., 2025).

Acute appendicitis is classified into two categories: simple (uncomplicated or non-perforated) and complicated (perforated). Thus, it is essential to assess the effectiveness of various treatment approaches for these two forms of appendicitis (Salminen et al., 2025). The mildest form of appendicitis, known as suppurative or phlegmonous appendicitis, is marked by a substantial infiltration of neutrophils into the muscular wall of the appendix. This variant is commonly termed uncomplicated or simple appendicitis (SA). In contrast, complicated appendicitis (CA) involves inflammation accompanied by vascular thrombosis, leading to necrosis of the appendix wall—gangrenous appendicitis. If the wall eventually ruptures, luminal contents leak, producing perforated appendicitis, which can precipitate a complex intra-abdominal infection. Such an infection may present as diffuse peritonitis or as a localized periapical abscess (Gorter et al., 2016; Salminen et al., 2025).

A healthy appendix harbors a robust and diverse community of microorganisms, such as Bacillota, Bacteriodota, Actinomycetota, and Pseudomonadota. Studies show that the gut microbiota of patients with appendicitis differs from that of healthy controls. The microbial profile also varies between SA and CA. An appendectomy has been noted to temporarily diminish gut bacterial diversity (Salminen et al., 2025). Changes in the microbiota associated with appendicitis are known to significantly influence the pathophysiology of AA. Therefore accurately distinguishing between SA and CA cases is essential for selecting suitable treatment approaches. This study aimed to investigate microbial differences between SA and CA in children’s appendix tissue and rectal swab samples through culture and microbiota analyses, and to evaluate how these differences influence treatment decisions. Due to the absence of a completely healthy control group, appendix tissue samples were compared between the two groups.

## 2 Material and methods

### 2.1 Study design

This single-center prospective cohort study encompasses pediatric patients who presented to the Pediatric Surgery Department with acute abdominal complaints between July 2025 and March 2026. Of the 129 patients who underwent appendectomy for AA during this period, only 39 (30%) samples were included in the study, excluding weekend operations. The small sample size may be attributed not only to the exclusion of patients who presented outside of working hours or were immunosuppressed, but also to the inability to perform surgeries following the earthquake and the failure to collect samples due to a serious health issue experienced by the project coordinator. Four patients were excluded from the study due to library preparation failure or very small 16S amplicon library size, but the study continued with the addition of further appendicitis patients.

### 2.2 Ethical approvement

The Istanbul Faculty of Medicine Clinical Research Ethics Committee approved the study (14.04.2023/08 - 20.09.2024/18). The study was performed in accordance with the Declaration of Helsinki. Informed consent was obtained from each child’s parents prior to the procedure.

### 2.3 Patients and healthy volunteers

The study enrolled children aged 2 to 18 years who were suspected of AA based on history and physical examination. Inclusion required a confirmed diagnosis of AA, evidenced by clinical symptoms such as abdominal pain, nausea, and vomiting, elevated inflammatory markers (e.g., WBC, CRP), imaging findings (X-ray, ultrasound, CT), and histopathological confirmation after appendectomy. Exclusion criteria were prior abdominal surgery, other gastrointestinal diseases such as inflammatory bowel disease, and immunocompromised conditions including malignancy. Disease severity was evaluated using the Pediatric Appendicitis Score (PAS) and the Alvarado score (Salminen et al., 2025).

Healthy children undergoing unrelated procedures, including circumcision and surgery for undescended testis, were recruited as controls for rectal swab microbiota analysis. Rectal swab collection did not require an additional invasive surgical procedure. In contrast, obtaining appendiceal tissue from healthy children solely for research purposes was not considered ethically justified. It is known that without an appropriate inflamed tissue healthy control group, all comparisons of tissue-derived microbiota with normal appendix microbiota will remain purely descriptive. Therefore, only one non-inflamed appendiceal tissue specimen, obtained during an incidental appendectomy in a child without appendicitis, was available as a descriptive tissue reference. Because a single specimen cannot constitute an adequate statistical control group, inferential analyses of appendiceal tissue microbiota were restricted to comparisons between SA and CA.

### 2.4 Surgery procedures

All appendectomy operations were performed by the same clinician. Ampicillin + sulbactam was administered prophylactically to SA group patients 30 minutes before appendectomy, and ampicillin + sulbactam and metronidazole were administered to patients with perforated appendicitis. Appendiceal tissue and rectal swab samples were obtained during appendectomy from patients. During operation, the diseased appendix was removed. After excising the vermiform appendix, a fragment of roughly 1 cm^3^ from the proximal end was cut with a sterile scalpel, placed in a sterile screw-cap container, and immediately transferred to medical microbiology laboratory. The rest of the appendix specimen was sent to pathology laboratory for routine histopathologic examination.

Rectal swab samples were obtained from children with appendicitis and from healthy children undergoing unrelated procedures. These healthy children constituted the control group for rectal microbiota comparisons. The single non-inflamed appendiceal tissue specimen was used only as a descriptive tissue reference and was not treated as a statistical control group.

### 2.5 Microbiological tests

Appendix specimens collected during surgery were placed in sterile screw-capped containers and divided into two aliquots. One aliquot was stored in a deep freezer at -80°C for subsequent microbiota analysis, while the other was immediately inoculated into thioglycolate broth and incubated at 37 °C for 48 h. After this period, both aerobic and anaerobic cultures were performed. For aerobic culture, appendix tissue and rectal swab specimens was inoculated onto 5% sheep blood agar and Chromagar Orientation medium and incubated at 37 °C K for 24–48 h in an aerobic atmosphere. At the end of incubation, the bacteria isolated were identified with an automated identification system (VITEK-2, bioMerieux, France) and antibiotic susceptibility testing by disk diffusion method was carried out. For anaerobic culture, the specimens was plated onto Schaedler agar and incubated at 37 °C for 2–5 days in anaerobic environment. Anaerobes were identified with an automated identification system (VITEK-2, bioMerieux, France). Antibiotic susceptibility tests were carried out according to European Committee on Antimicrobial Susceptibility Testing (EUCAST) recommendations (EUCAST, 2024).

### 2.6 16S rRNA sequencing

Only children with histopathologically confirmed appendicitis were included in final microbiota analysis. Tissue samples were prepared using the DiaRex^®^ Tissue Genomic DNA Extraction Kit (Ankara, Turkiye). DNA extraction from rectal swab samples was performed utilising Stool Genomic DNA Extraction Kit (Ankara, Turkiye). The 16S rRNA regions were amplified using a DNA preparation kit with primers designed to target the V3-V4 region. Microbiota analysis were performed at phylum, order, class, family and genus levels.

### 2.7 Bioinformatic analysis

16S rRNA sequencing data were processed using QIIME 2 (v2025.7). Single-end Phred33 reads were quality-filtered, denoised, and processed for chimera removal and ASV inference using the DADA2 denoise-single workflow. Reads were truncated at 145 bp without 5′ trimming (trunc_len = 145; trim_left = 0), with a maximum expected error threshold of 2.0 (max_ee = 2) and a quality truncation threshold of 2 (trunc_q = 2). Chimeric sequences were removed using the consensus method. Following DADA2 processing, sequencing depth ranged from 535 to 688,304 non-chimeric reads per sample (median: 173,710). Taxonomic classification was performed against the SILVA 138.2 SSURef NR99 database through the genus level.

For diversity analyses, the ASV table was aggregated at the genus level. Genus-level count depth ranged from 200 to 247,737 counts per sample (median: 52,736). For alpha-diversity analysis, the count matrix was rarefied to 2,024 counts per sample, corresponding to the floor of the 5th percentile of the sequencing-depth distribution. Rarefaction was applied specifically for alpha-diversity comparisons to standardize sequencing depth across samples and reduce the influence of unequal library sizes on within-sample diversity estimates. Shannon, Simpson, and observed genera were calculated using phyloseq and compared using the Kruskal–Wallis test followed by Dunn’s test with Benjamini–Hochberg correction.

For beta-diversity analysis, genus-level counts were converted to relative abundances without rarefaction. Bray–Curtis dissimilarities were calculated and visualized using PCoA, and differences in microbial community composition were assessed by PERMANOVA using adonis2 in the vegan package. Normality was assessed using the Shapiro–Wilk test, followed by pairwise Wilcoxon tests with FDR correction. Visualizations were generated using ggplot2.

Differential abundance at the genus level was additionally assessed using ANCOM-BC2 on the original non-rarefied count data to account for the compositional nature of microbiome data and sampling-fraction bias. Genera present in fewer than 10% of samples were excluded, and samples with library sizes below 1,000 counts were removed from this analysis. Benjamini–Hochberg correction was applied for multiple testing, with q < 0.05 considered statistically significant. Tissue analysis was restricted to SA and CA groups because only one healthy appendiceal tissue control was available.

## 3 Results

A total of 39 patients and 11 controls were included in this study. Demographic information for patients and controls was obtained from the Hospital and Laboratory Information Systems.

### 3.1 Demographic data of patients

Of the patients, 24 were boys and 15 were girls. The mean age in the patients’ groups was 11.65 (min-max: 5-17), while in the control group it was 9 (min-max: 2-17) (**Table 1**). Ninety-five percent of the patients had a Glasgow Coma score of 15. Based on the histopathological examinations, 16 (41%) of the 39 patients were classified as CA and the rest as SA. Six (37.5%) of the CA cases suffered from perforated appendicitis, and 10 (62.5%) had periapical abscesses. Bacterial growths were also detected in the peritoneal fluid of perforated appendicitis cases.

**Table 1:** Demographic characteristics, clinical and laboratory parameters of patient and control groups.

PATIENTS	HC group (n=11)	SA group (n=23)	CA group (n=16)	p value (SA-HC)	p value (CA-HC)	p value (SA-CA)
Gender (boys/girls)	8/3	15/8	9/7	0.34	0,20	0.29
Mean age (years)	9.00 ± 6.06	11.35 ± 3.94	12.00 ± 3.20	0.09	**0,05**	0.29
WBC (10^3^/uL)	8.85 ± 2.7	16.62 ± 4.72	16.72 ± 4.95	**<0.001**	**<0.001**	0.95
RBC (10^6^/uL)	4.61 ± 0.63	4.90 ± 0.49	4.78 ± 0.44	0.08	0.21	0.22
Hb (g/dL)	12.62 ± 0.77	13.18 ± 1.52	13.236 ± 1.31	0.14	0.09	0.46
Htc (%)	36.92 ± 3.34	38.84 ± 4.06	38.82 ± 3.22	0.09	0.08	0.49
CRP (mg/L)	0.68 (0.5-14.5)	10.67 (0.5-59)	35.9 (1.39-279.65)	**0.004**	**0.001**	**0.002**
PCT (ng/mL)	0.27 (0.05-0.39)	0.24 (0.03-1.23)	0.34 (0.18-7.18)	0.90	0.42	0.09
ALT (U/L)	18.3 (11-45)	14.46 (8.6-80)	16.3(13-50)	0.58	0.42	0.19
AST (U/L)	25.5 (13.3-42.7)	18.9 (9.4-31.8)	18.2 (8.5-56.4)	0.13	**0.016**	0.99
**Creatinine** (mg/dL)	0.51 ± 0.16	0.60 ± 0.13	0.63±0.14	0.16	0.11	0.50

HC, Healthy control group; SA, Simple appendicitis group; CA, Complicated appendicitis group; WBC, White Blood Cell; RBC, Red Blood Cell; Hb, Hemoglobin; Htc, Hematocrite; CRP, C-Reactive Protein; PCT, Procalcitonin; ALT, Alanine aminotransferase; AST, Aspartate aminotranferase.

Numbers in bold indicate those that are statistically significant.

When the SA and CA groups were compared with the control group, significant differences were found in WBC and CRP levels; however, when the SA and CA groups were compared with each other, no significant difference was found in WBC levels. A statistically significant difference was found, when the AST levels of the CA group were compared with those of the control group (**Table 1**, **Figure 1**).

**Figure 1:**
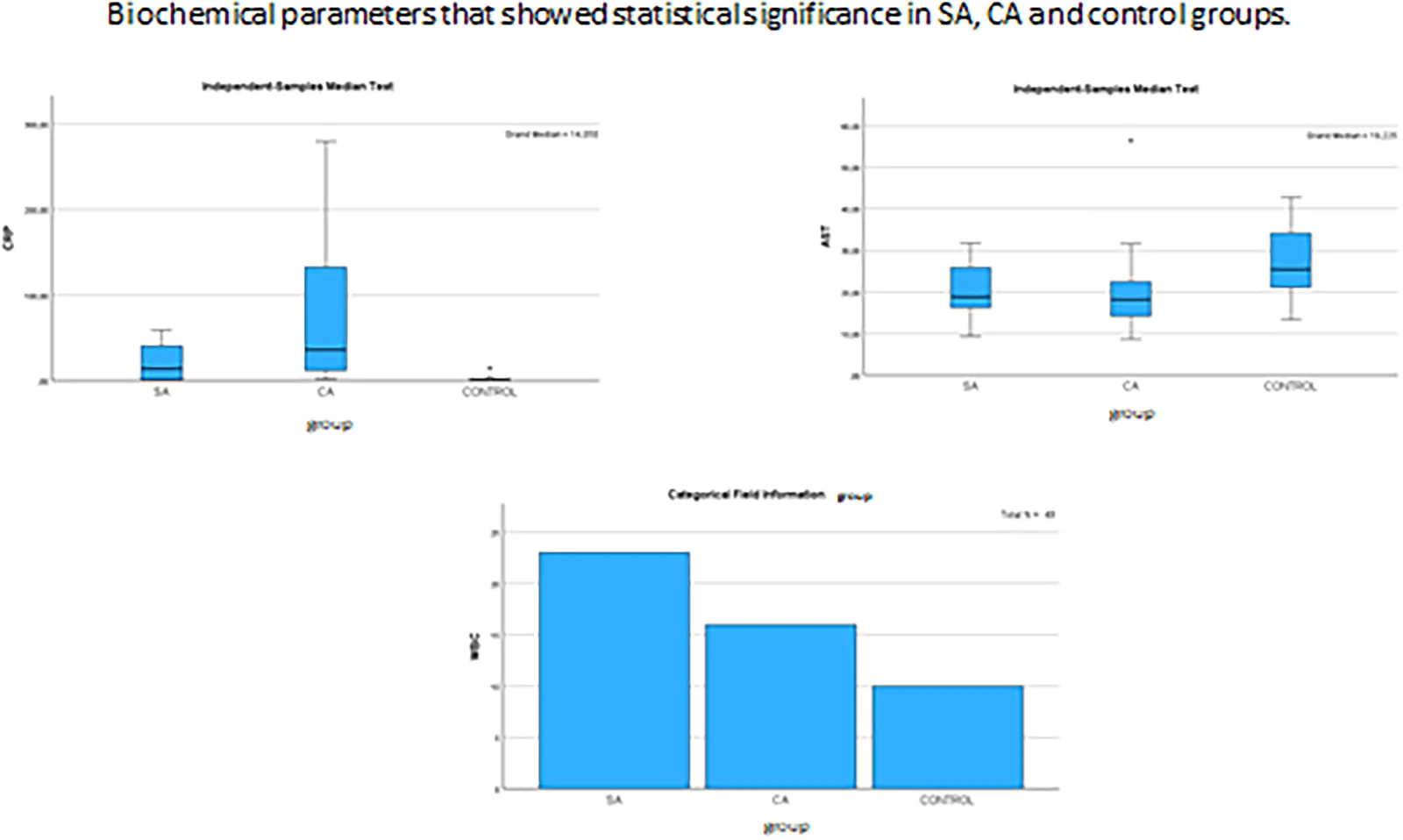
Biochemical parameters that showed statistical significance in SA, CA and control groups.

### 3.2 Results of conventional culture

Based on conventional culture, the most common aerobic bacteria in rectal and tissue samples from groups SA and CA were *Escherichia*, *Klebsiella* spp., and *Enterococcus* spp. Gram-negative bacilli producing ESBL were isolated from 48% of patients in the SA group and 31% of patients in the CA group. This rate was lower (9%) in the control group. The anaerobic bacteria isolated varied depending on the groups and sample types. In rectal samples from SA and CA groups, the most common anaerobic bacteria were *Lactobacillus* spp.(13%) and *Peptostreptococcus* spp. (13%); Clostridium spp. (12.5%) and *Prevotella* spp.(12.5%). In tissue samples from SA and CA groups, the most common bacteria were *Anaerococus* spp. (13%); *Bacteroides* spp. (12.5%), and *Fusobacterium* spp. (12.5%), respectively (**Table 2**).

**Table 2:** The most predominant (rate of >10%) genera in rectal and tissue samples of the SA and CA group using conventional culture.

Rectal swabs	SAR group	CAR group	HCR group	Appendiceal tıssue	SAT group	CAT group
Aerobic	*Escherichia* spp.	*Escherichia* spp.	*Escherichia* spp.	Aerobic	*Escherichia* spp.	*Escherichia* spp.
	*Klebsiella* spp.	*Klebsiella* spp.	*Enterococcus* spp.		*Klebsiella* spp.	*Klebsiella* spp.
	*Enterococcus* spp.	*Enterococcus* spp.	*Klebsiella* spp.		*Enterococcus* spp.	*Enterococcus* spp.
	*Proteus* spp.	*Staphylococcus*				*Pseudomonas* spp.
Anaerobic	*Lactobacillus* spp.	*Clostridium* spp.	*Clostridium* spp.	Anaerobic	*Anaerococcus* sp.	*Fusobacterium* spp.
	*Peptostreptococcus*	*Prevotella* spp.	*Peptostreptococcus*			*Bacteroidees* spp.
			*Peptoniphilus* spp.			

SAR, Simple appendicitis-rectal swab; SAT, Simple appendicitis-appendiceal tissue; HCR, Healthy control- rectal swab; CAR, Complicated appendicitis-rectal swab; CAT, Complicated appendicitis-appendiceal tissue.

### 3.3 Results of microbiota analysis

No statistically significant distinction was shown at the alpha and beta diversity levels between SA and CA cases (**Figure 2a** and **3a**). Pseudomonadota (prev. Proteobacteria), Bacillota (prev. Firmicutes), and Bacteriodota (prev. Bacteroidetes) and Fusobacteriota were the most common in tissue samples, while Bacillota, Pseudomonadota, Bacteriodota and Actinomycota phylums were predominant in rectal samples (**Figure 2b** and **3b**). The phyla whose abundance increased and decreased in tissue and rectal samples compared to the control groups is indicated in **Table 3**. **Figure 4** shows the more abundant genera in SA and CA groups compared to the control group in rectal swab samples. The relative abundances of rectal swab samples at phylum and genera levels are indicated in **Figures 5** and **6**.

**Figure 2:**
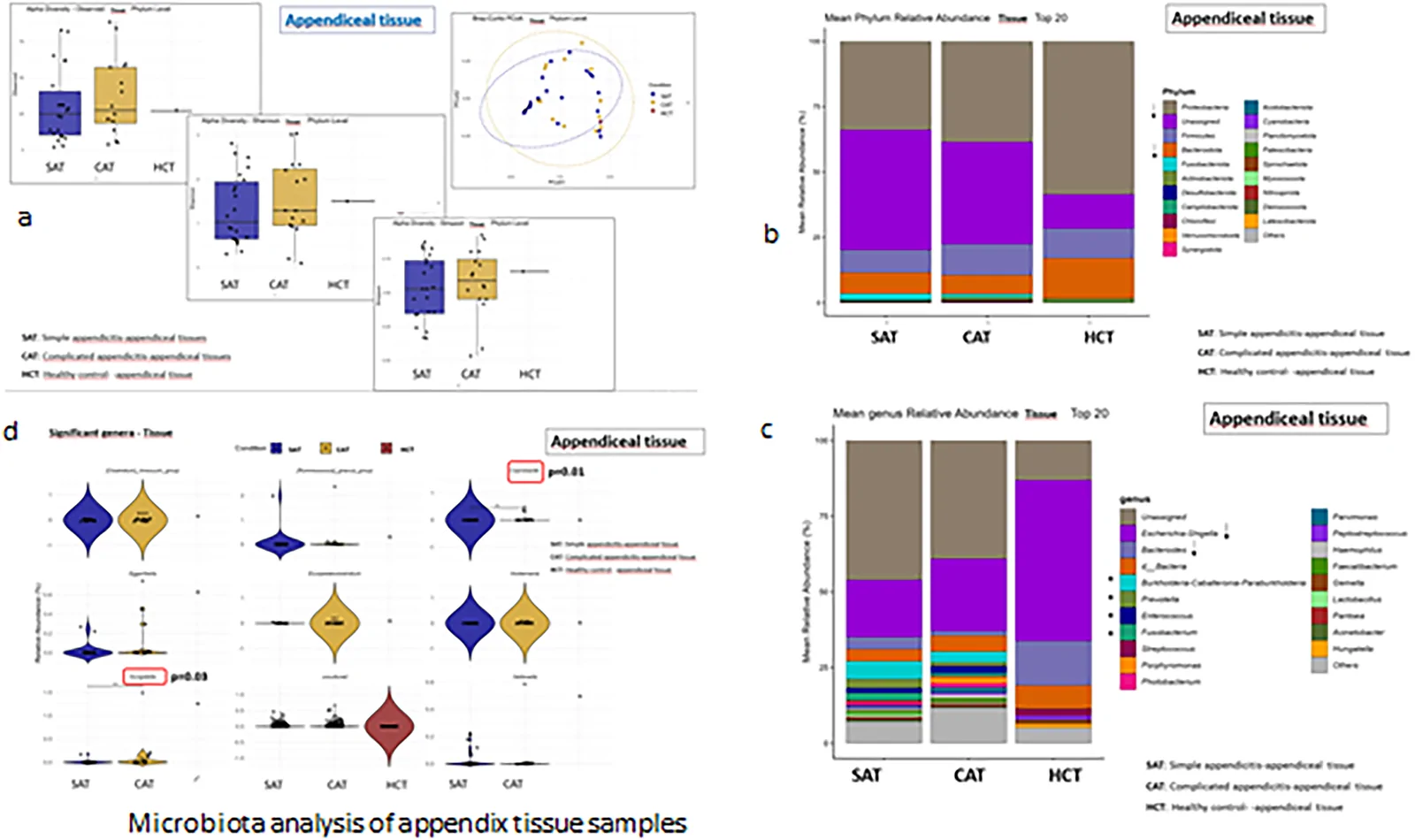
Microbiota analysis of appendix tissue samples: **(a)** Alpha diversity (observed, Simpson, Shannon) and beta diversity (Bray-Curtis-PCoA) analyses, **(b)** Mean relative abundance - Phylum level, **(c)** Mean relative abundance - Genus level, **(d)** Genera showing statistically significant differences between SA and CA groups.

**Figure 3:**
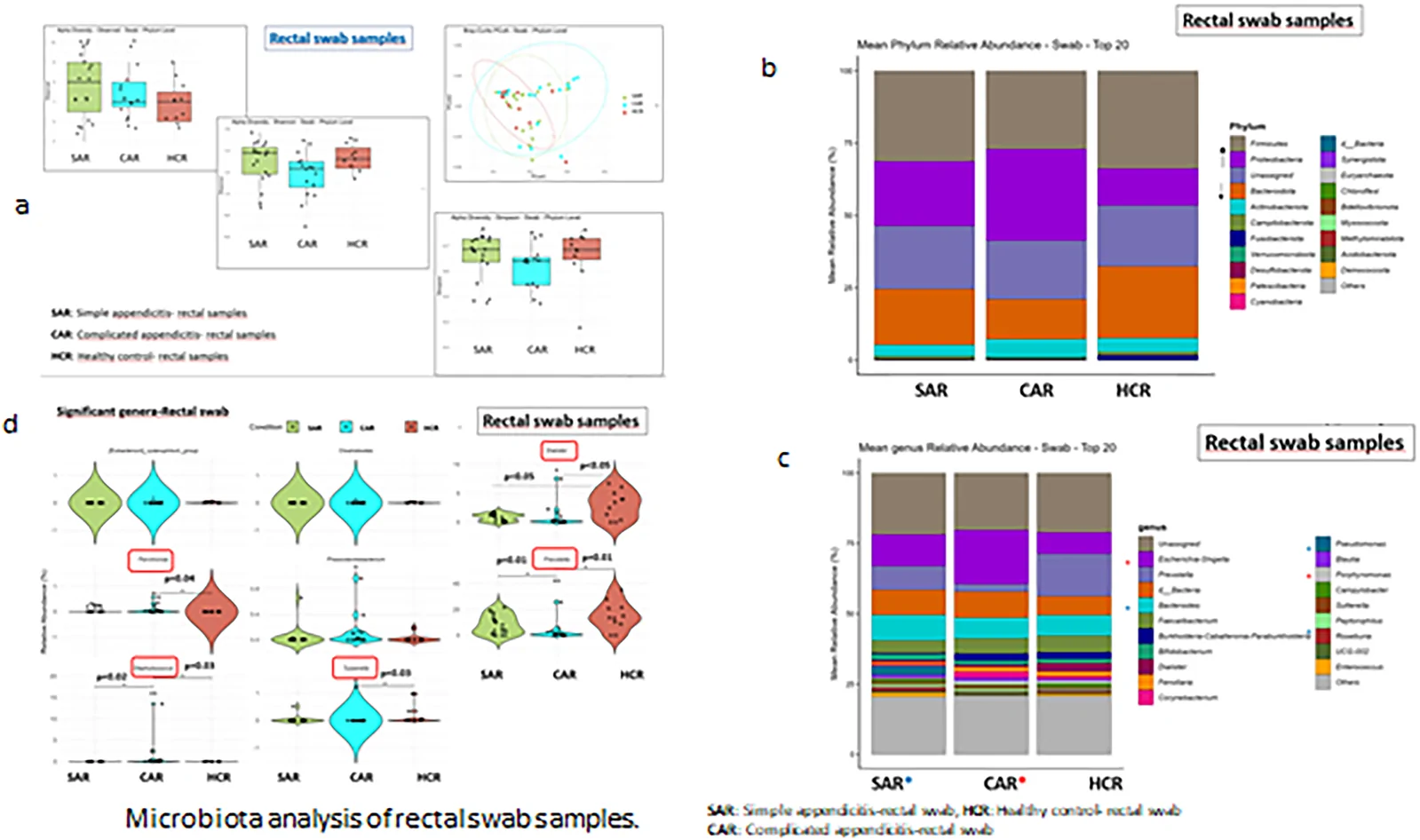
Microbiota analysis of appendix rectal samples: **(a)** Alpha diversity (observed, Simpson, Shannon) and beta diversity (Bray-Curtis-PCoA) analyses, **(b)** Mean relative abundance - Phylum level, **(c)** Mean relative abundance - Genus level, **(d)** Genera showing statistically significant differences between SA and CA groups.

**Table 3:** Phyla that increased and decreased in tissue and rectal samples compared to the control group.

Phylums	Rectal swabs (%)				Tissue samples (%)		
	SAR	CAR	HCR	Increase/ Decrease (SAR vs. HCR/CAR vs. HCR)	SAT	CAT	Increase/Decrease (SAT vs. CAT)
Firmicutes	31.2	27	33.7	Decrease/ Decrease	8.59	11.91	Increase
Bacteroidetes	19.2	13.7	24.8	Decrease/ Decrease	8.02	7.28	Decrease
Actinobacteria	3.9	6.32	4.75	Decrease/ Increase	0.48	1.38	Increase
Fusobacteria	0.19	0.22	1.63	Decrease/ Decrease	2.43	0.99	Decrease
Proteobacteria	22.3	31.7	12.73	Increase/ Increase	33.84	38.49	Increase
Firmicutes/Bacteroidetes	1.62	1.97	1.36	Increase/ Increase	1.07	1.63	Increase

SAR, Simple appendicitis-rectal swab; SAT, Simple appendicitis-appendiceal tissue; HCR, Healthy control- rectal swab; CAR, Complicated appendicitis-rectal swab; CAT, Complicated appendicitis-appendiceal tissue.

**Figure 4:**
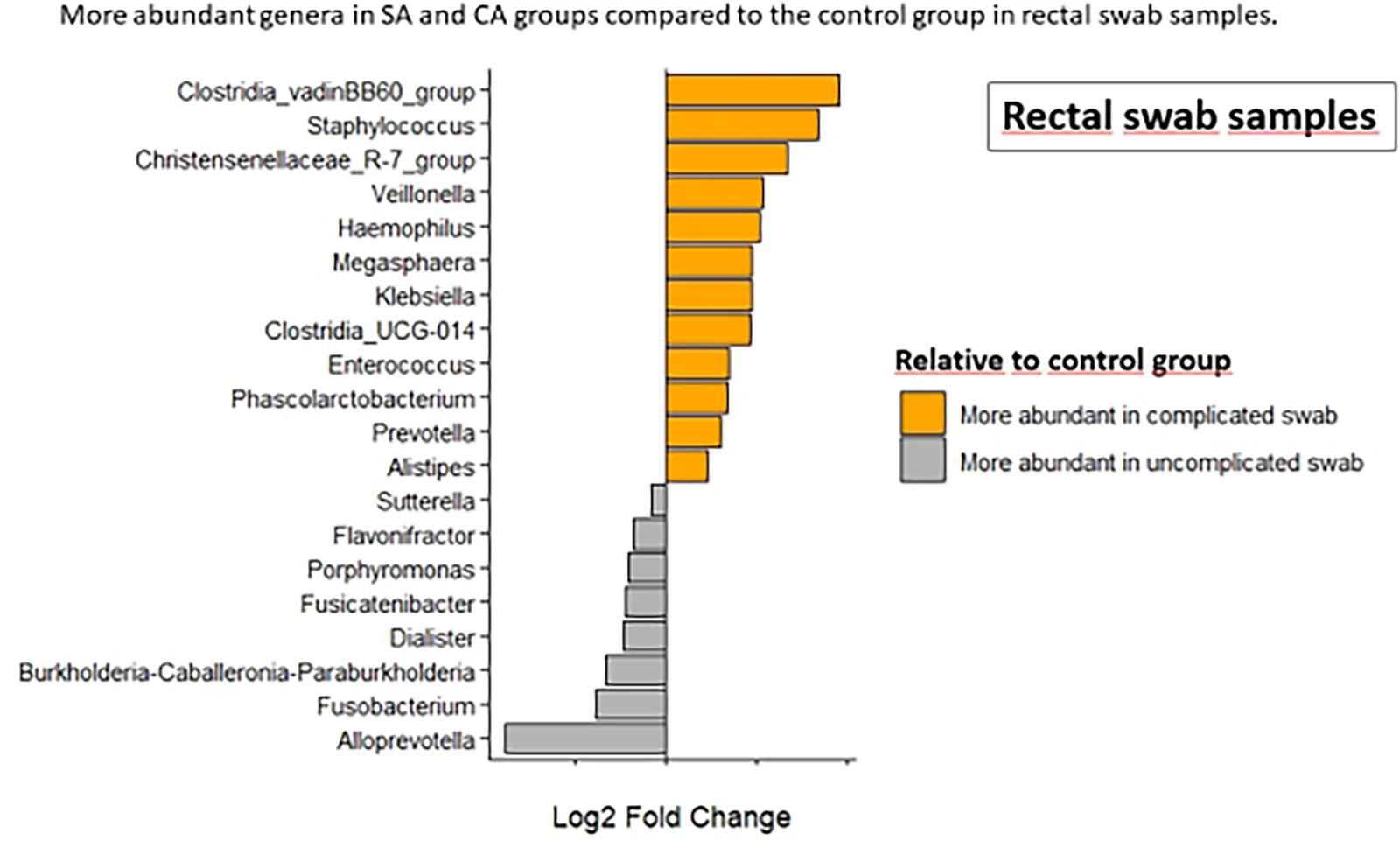
More abundant genera in SA and CA groups compared to the control group in rectal swab samples.

**Figure 5:**
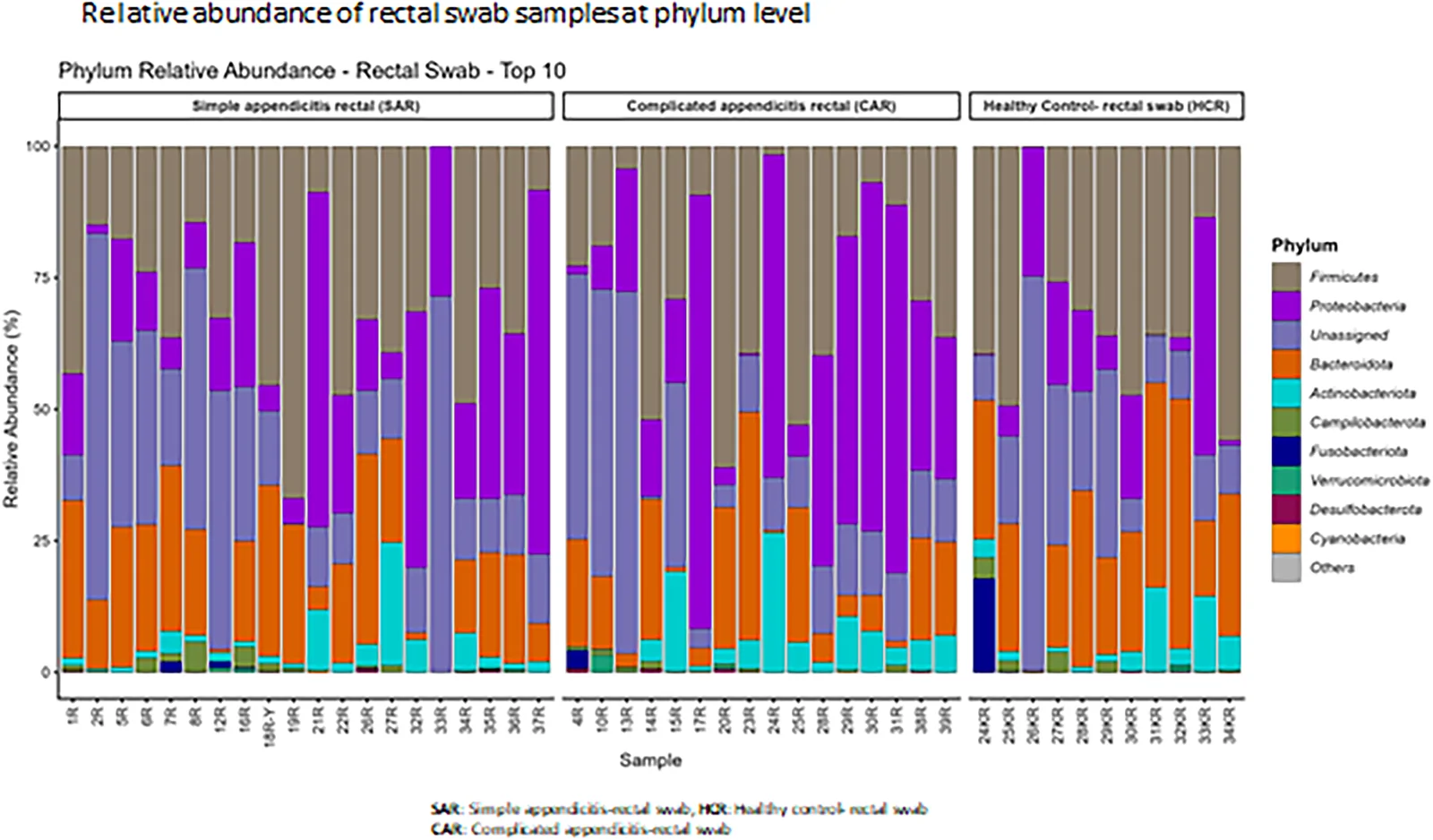
Relative abundance of rectal swab samples at phylum level.

**Figure 6:**
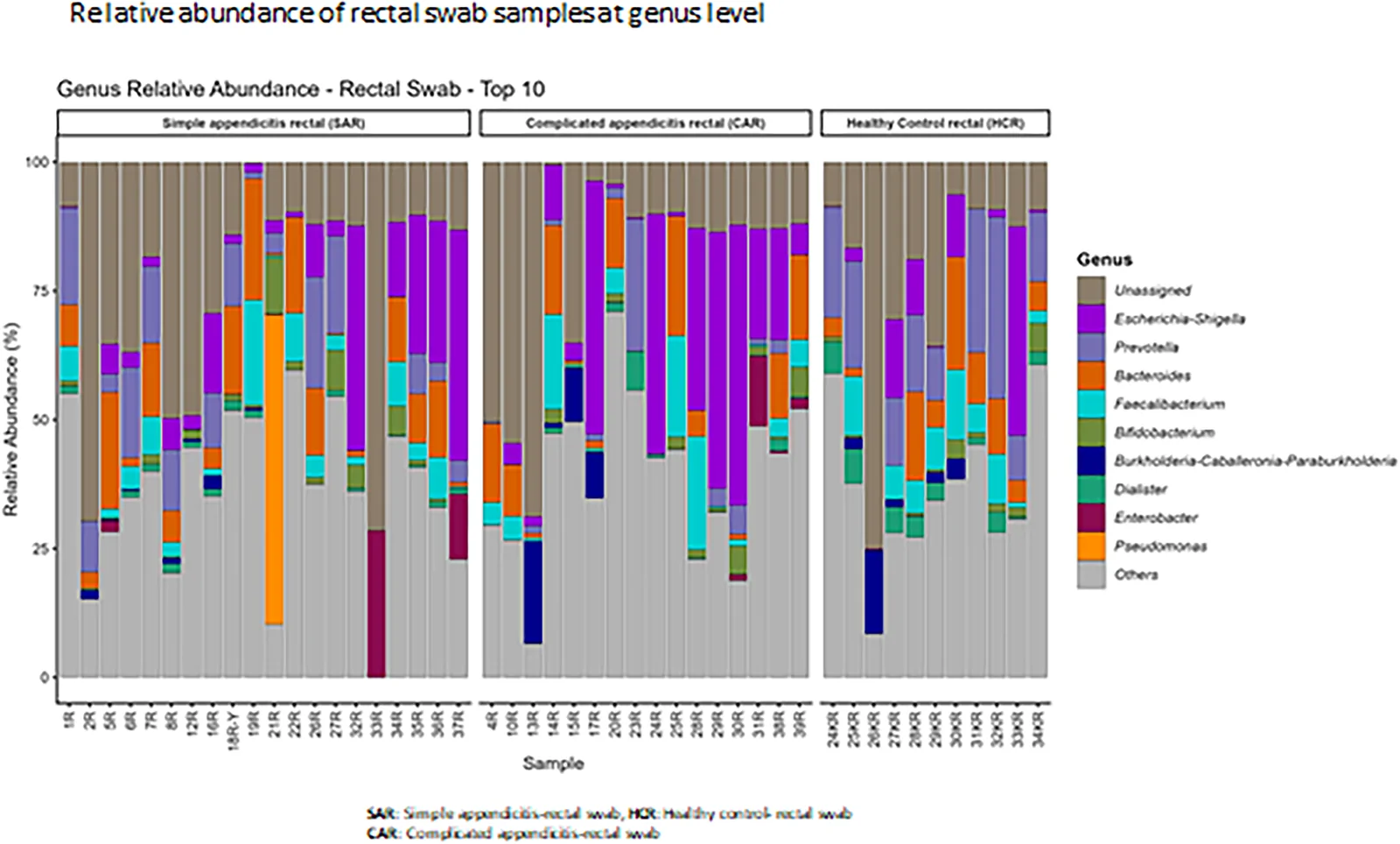
Relative abundance of rectal swab samples at genus level.

The raw 16S rRNA gene sequencing data generated in this study are available in the European Nucleotide Archive (ENA) under the project accession number PRJEB126667 (ERP206277). The datasets analyzed for this study can be found in the [PRJEB126667 (ERP206277).] [https://www.ebi.ac.uk/ena/browser/home].

## 4 Discussion

Research on microbiota profiling in SA and CA has increased in recent years to better understand disease pathogenesis and to evaluate the potential contribution of microbiota to clinical decision-making. In the present study, although several taxa differed between SA and CA at the raw p-value level, no genus remained significantly different after multiple-testing correction in either appendiceal tissue or rectal swab samples. This should not be interpreted as biological equivalence between the groups. Rather, the relatively small sample size, particularly in the CA group, may have reduced statistical power and increased the likelihood of Type II error.

### 4.1 Patients

The predominance of boys and the older age of patients with CA were consistent with previous reports suggesting that the risk of CA increases with age in children. WBC and CRP levels were significantly higher in both appendicitis groups than in controls, in agreement with Liu et al. (2025). Although WBC values did not differ between SA and CA, CRP levels were significantly higher in CA, reflecting more advanced inflammation and supporting previous findings (Aiyoshi et al., 2025). Hematological parameters (RBC, Hgb, Htc) showed no significant differences among groups, while AST, but not ALT or creatinine, was significantly higher in CA (**Table 1**).

### 4.2 Conventional culture

As expected, conventional culture detected fewer microorganisms than microbiota analysis because many intestinal bacteria, particularly anaerobes, cannot be cultured under routine laboratory conditions (Sarah-May et al., 2019). Culture yields reflect the live organisms that can be recovered under laboratory conditions and do not represent the absolute microbial loads present *in situ*. In our study, delayed transport limited the recovery of anaerobic bacteria, resulting in no anaerobic growth in 35% of samples. Consequently, culture-based findings likely underestimated the true microbial diversity, and culture results and microbiota analysis results are not directly comparable.

Despite these limitations, culture and microbiota analyses were generally consistent at the phylum level, although this differs from the findings of Schülin et al. (2017). Culture results also suggested a more stable microbial composition in controls, whereas appendicitis samples showed reduced diversity or expansion of opportunistic pathogens depending on disease severity (**Table 2**).

*Escherichia, Klebsiella*, and *Enterococcus* species remained the dominant cultured organisms regardless of disease severity or specimen type, confirming their role as common intestinal flora (**Table 2**). Anaerobic bacteria, however, varied according to appendicitis severity. Although increases in *Klebsiella* spp. and *Pseudomonas* spp. in rectal swabs were not statistically significant, the high prevalence of ESBL-producing isolates in SA (48%) and CA (31%) is clinically noteworthy and may influence future prophylactic antibiotic strategies. These rates exceed those reported by Snyder et al. (16–37%) (Snyder et al., 2024), highlighting the need for larger prospective studies. When all (SA+CA) patients were evaluated based on isolated bacteria, the ESBL positivity rate in *E. coli* and *Klebsiella* isolates isolated from culture was found to be 31.8% and 16.7%, respectively These rates were similar to the ESBL positivity rates (21-56.8%) in *E. coli* strains isolated from clinical samples in our country, but were lower than the rates in *Klebsiella* isolates (28.7-65.1%) (Şahin and Göçer, 2025; Akıllı, 2025; Bayraktar and Açıkgül, 2025; Yıldırım et al., 2025).

ESBL positivity was observed in both SA and CA groups, but its distribution varied according to bacterial species and specimen type rather than showing a consistent association with disease severity. In appendix tissue, ESBL-producing *E. coli* was detected more frequently in the SAT group than the CAT group (39.0% vs. 16.7%), while ESBL-producing *Klebsiella* spp. showed the opposite pattern (0% vs. 33.3%). Similarly, in rectal swabs, the ESBL positivity rates for *E. coli* were 25.0% and 18.75% in the SAR and CAR groups, respectively, while the corresponding rates for *Klebsiella* spp. were 8.3% and 11.1%, respectively. In other words, in both sample groups, ESBL positivity was observed to decrease in *E. coli* isolates and increase in *Klebsiella* isolates in the CAR group compared to the SA group. These findings suggest that ESBL carriage (or detection) in appendix samples cannot be directly correlated with disease severity, but rather may reflect differences in the distribution of antimicrobial-resistant Enterobacterales among individual patients. While the presence of ESBL-producing isolates in rectal samples may represent the intestinal reservoir, their detection in appendix tissue suggests their presence in the local microbial environment during AA. However, a direct relationship or clonal identity between the two populations cannot be established. Molecular typing or whole-genome sequencing is necessary to determine whether resistant isolates from two different anatomical regions represent the same strains.

The bacterial spectrum identified in CA group, including *Escherichia*, *Klebsiella, Enterococcus, Staphylococcus, Bacteroides, Prevotella, Fusobacterium*, and *Clostridium species* (**Table 2**), was consistent with previous reports. Likewise, studies from Türkiye identified *Escherichia, Klebsiella, and Enterococcus* as the predominant pathogens in appendicitis (Tartar et al., 2015; Grassie et al., 2020). Kakar et al. additionally reported frequent isolation of *E. coli* and *P. aeruginosa* in CA (Kakar et al., 2022). Similarly, in a study examining the lumen contents of the appendix of 694 patients who underwent appendectomy due to AA, the most common microorganisms were *E. coli* (65%) and *P. aeruginosa* (16%) (Song et al., 2018). Our findings also emphasized the presence of *Klebsiella* and *Enterococcus*. The coexistence of anaerobic genera such as *Fusobacterium* and *Prevotella* together with dominant aerobic bacteria may reflect increasing microbial complexity during disease progression.

Consistent with previous culture-based studies, aerobic bacteria showed little difference between SA and CA, whereas changes in anaerobic communities appeared more closely associated with disease severity (Hattori et al., 2019). Overall, these findings support the importance of anaerobic dysbiosis in appendicitis progression while also demonstrating the limitations of conventional culture in accurately characterizing the appendiceal microbiota.

Although PERMANOVA comparisons were not statistically significant, the effect size was slightly greater in rectal swab samples (R² = 0.059, p = 0.139) than in appendiceal tissue samples (R² = 0.049, p = 0.500), suggesting that disease status explained a modest proportion of the microbial variation. However, this finding should not be interpreted as evidence of diagnostic utility because sensitivity, specificity, ROC/AUC, and predictive performance were not evaluated, and ANCOM-BC2 identified no significant genus-level differences between SA and CA after multiple-testing correction. Therefore, rectal swabs should currently be regarded as a non-invasive sampling method for microbiota research rather than a diagnostic tool for differentiating appendicitis severity. This finding is consistent with Sarah-May et al., who also reported limited diagnostic performance of rectal microbiota (Sarah-May et al., 2019).

### 4.3 Microbiota analysis

In both appendiceal tissue and rectal samples, Bacillota, Pseudomonadota, and Bacteriodota were the predominant phyla, consistent with previous reports showing that the overall phylum composition remains relatively stable in appendicitis (Oh et al., 2020; Islam et al., 2025). Although some studies have found significant differences between SA and CA groups (Jackson et al., 2014; Salö et al., 2017; Sarah-May et al., 2019; Vanhatalo et al., 2022; Aiyoshi et al., 2023; Liu et al., 2025), other studies, including this one, have shown no difference between the two groups (Peeters et al., 2019). In the present study, the Firmicutes/Bacteroidetes ratio increased in both sample types, particularly in CA (**Table 3**), suggesting progressive microbial dysbiosis despite relatively stable overall diversity.

Previous studies have reported inconsistent microbial signatures associated with SA and CA. In our cohort, the predominance of *Burkholderia* in SA and *Prevotella* in CA agreed with Liu et al. (2025), whereas the enrichment of *Fusobacterium* in SA and *Veillonella* in CA was consistent with Ma et al. (2025). Similarly, the dominant phyla identified by Guinane et al. (2013) resembled our findings and further supported the involvement of oral-associated bacteria such as *Fusobacterium, Parvimonas*, and *Gemella* in appendicitis. In contrast, Sarah-May et al. (2019) and Bi et al. (2022) reported different dominant taxa, while Schülin et al. (2017) found *Fusobacterium* and *Porphyromonas* to predominate in CA.

Unlike many previous studies reporting *Fusobacterium* enrichment in CA (Guinane et al., 2013; Zhong et al., 2014; Schülin et al., 2017; Vanhatalo et al., 2022; Aiyoshi et al., 2023; Liu et al., 2025; Karikis et al., 2026), *Fusobacterium* was not the predominant genus in our CA group. Instead, CA appeared to be characterized by polymicrobial dysbiosis involving several anaerobic taxa rather than a single dominant organism. This observation is supported by studies demonstrating alternative microbial profiles in CA (Sarah-May et al., 2019; Bi et al., 2022; Ma et al., 2025).

The discrepancies among studies evaluating the microbiota of SA and CA may be attributable to differences in patient populations, disease severity, sampling sites and procedures, as well as sequencing and bioinformatic approaches. Previous pediatric studies have reported contrasting findings regarding the association between microbial composition and appendicitis severity, with some demonstrating increased microbial diversity and abundance in CA cases (Sarah-May et al., 2019), whereas others have found substantial within-group variability and no clear association with the histological grade of inflammation (Salö et al., 2017). Moreover, microbiota profiles may vary according to the sampling site, as distinct microbial compositions have been reported in appendiceal luminal, mucosal, extraluminal, and rectal samples (Schülin et al., 2017; The S-MM et al., 2022). Methodological differences, including the 16S rRNA region analyzed, sequencing platform, DNA extraction procedures, and bioinformatic pipeline, may further contribute to differences in microbial diversity and relative taxonomic abundance across studies (Allali et al., 2017; Gibbons et al., 2018). Finally, geographical and environmental factors can influence the composition of the gut microbiota, potentially limiting direct comparisons between populations from different regions (Kemppainen et al., 2015). Our findings should therefore be interpreted in the context of these methodological and population-level differences. Collectively, the available evidence suggests that although oral-associated anaerobes play an important role in appendicitis, no universal microbial signature consistently distinguishes SA from CA.

One important factor influencing microbiota analysis findings may be pre-operative antibiotic exposure. Patients with perforated appendicitis received metronidazole in addition to ampicillin+sulbactam, whereas patients with SA received only ampicillin+sulbactam. No pre- antibiotic samples were collected. A single prophylactic antibiotic dose given 30 minutes before surgery may have a more limited effect on the microbiota compared to long-term antibiotic treatment. Nevertheless, the fact that sampling was performed after antibiotic administration may have led to some differences in the detected anaerobic taxa. Because metronidazole has potent activity against anaerobic bacteria, including *Fusobacterium, Prevotella, Parvimonas*, and *Porphyromonas*, differences in these taxa may partly reflect antimicrobial exposure rather than disease severity alone. As it is impossible to disentangle disease-driven dysbiosis versus antibiotic-driven microbiota perturbation in the current study design, the microbiota differences observed between SA and CA should be interpreted as the combined effects of disease-associated dysbiosis and pre-operative antibiotic treatment.

#### 4.3.1 Tissue samples

Compared with controls, both SAT and CAT samples showed reduced abundances of Pseudomonadota and Bacteriodota together with marked genus-level shifts (**Figure 2b**). While controls were dominated by *Escherichia* and *Bacteroides*, appendicitis samples demonstrated increased abundances of *Prevotella, Fusobacterium, Burkholderia*, and *Enterococcus*. At the nominal level, *Hungatella* showed higher and *Coprobacter* lower relative abundance in CAT (**Figure 2c**) compared with SAT (**Figure 2d**). However, no genus remained significantly different between SAT and CAT after compositional differential-abundance analysis using ANCOM-BC2 with multiple-testing correction (q < 0.05).

Nevertheless, the enrichment of oral-associated anaerobes is broadly consistent with previous studies (Zhong et al., 2014; Aiyoshi et al., 2023; Liu et al., 2025), although the higher abundance of *Fusobacterium* in SAT rather than CAT highlights the heterogeneity of appendiceal microbiota across patient populations. Conversely, there is also a study reporting that *Fusobacterium* is more abundant in CA (Vanhatalo et al., 2022).

#### 4.3.2 Rectal swab samples

Since it was not ethically appropriate to obtain appendix tissue samples from healthy volunteers, rectal swab samples were collected from all patients and healthy volunteers. Although rectal swab samples do not fully reflect the appendicitis niche microbiota (Liu et al., 2026), they were used to compare microbiota analyses from patients and healthy volunteers. Rectal swabs showed decreased Bacteriodota and increased Pseudomonadota compared with controls (**Figure 3b**). SAR samples displayed a relatively balanced microbial profile, whereas CAR samples were characterized by increased *Escherichia* and reduced *Prevotella* (**Figure 3c**). Descriptive and nominal differences in the relative abundance of several genera, including *Parvimonas, Prevotella, Dialister, Tyzzerella*, and *Staphylococcus*, were observed across the rectal-swab groups. These findings should be considered exploratory because the corresponding pairwise differences did not remain significant after multiple-testing correction (**Figure 3d**). *Sutterella*, a genus with reported anti-inflammatory properties (Cao et al., 2025), was also reduced in appendicitis. Compositional differential-abundance analysis using ANCOM-BC2 identified significant overall differences among the three groups for *Prevotella, Haemophilus, Dialister, Lactobacillus, Parasutterella*, and an unclassified genus within *Enterobacteriaceae* (q < 0.05). However, pairwise analysis identified no significantly different genera between SAR and healthy controls or between CAR and SAR after multiple-testing correction. The only significant pairwise difference was an enrichment of the unclassified *Enterobacteriaceae* genus in CAR compared with healthy controls (LFC = 3.56, q = 0.003).

Finally, our findings agree with a recent systematic review showing reduced *Faecalibacterium, Blautia*, and *Lachnospiraceae*, together with increased *Escherichia, Pseudomonas*, and *Bacteroides* in appendicitis (Sagor et al., 2025). Overall, our results support the concept that appendicitis is associated with microbial dysbiosis characterized by shifts in the relative abundance of specific taxa rather than major alterations in overall microbial diversity.

The main strength of this study is the combined evaluation of appendiceal tissue and rectal swab samples using both conventional culture and 16S rRNA microbiota profiling. However, several limitations should be considered.

First, appendiceal tissue was sampled from the tip of the appendix, which may not represent whole-appendix microbiota. This may not be representative of the entire microbiota of the appendix, which could have resulted in sampling bias. In addition, only one non-inflamed appendiceal tissue specimen was available because obtaining healthy appendiceal tissue solely for research purposes was not ethically justified. Therefore, this specimen was used only as a descriptive reference, and tissue microbiota comparisons were limited to the SA and CA groups.

Second, the relatively small sample size, particularly the limited number of CA cases, reduced the statistical power of diversity and differential abundance analyses. Consequently, the absence of statistically significant differences after multiple-testing correction should not be interpreted as biological equivalence, as clinically relevant differences may have remained undetected because of Type II error. Accordingly, our findings should be regarded as exploratory and hypothesis-generating.

Another important limitation is pre-operative antibiotic exposure. Although all patients received prophylactic antibiotics before appendectomy, patients with perforated appendicitis additionally received metronidazole. Because metronidazole is active against anaerobic bacteria, including *Fusobacterium, Prevotella, Parvimonas*, and *Porphyromonas*, some microbiota differences between SA and CA may reflect antimicrobial exposure rather than disease severity alone. Nevertheless, a single prophylactic dose given 30 minutes before surgery is expected to have a more limited effect on the microbiota than prolonged antibiotic treatment, and a recent randomized study found no significant effect of preoperative cefuroxime plus metronidazole on perforation outcomes (Jalava et al., 2025). Future studies will, whenever feasible, obtain microbiological samples before antibiotic administration or standardize antibiotic exposure across study groups.

An additional limitation was that specimen collection and processing were restricted to regular laboratory working hours because of institutional working-hour regulations. Consequently, some clinical specimens obtained during nights and weekends could not be processed for the study. This may have introduced a potential selection bias and reduced the representativeness of the study population. Had it been possible to include all patients operated on during the study period, both the number of CA cases and the total number of patients could have increased, thereby enabling a better comparison between the groups. In addition, differences in the timing between specimen collection and processing may potentially affect microbiota composition because of preanalytical factors Potential confounding factors capable of altering rectal microbiota (preoperative fasting, stress, minor interventional effects) may be present in control group subjects and may have led to selection bias.; however, and these factors are - as is the case in any surgical study—are an inherent part of the study itself. All interventions involving the control group subjects were performed in accordance with current anesthesia guidelines. Nevertheless, standardized specimen collection, storage, and processing procedures were applied to all samples included in the study.

Finally, the predominance of abscess-forming rather than gangrenous appendicitis in our cohort may have influenced the observed microbial profiles. In addition, the use of 16S rRNA sequencing limited taxonomic resolution to the genus level. Future studies using larger cohorts and shotgun metagenomic sequencing are needed to better define microbial signatures associated with appendicitis severity.

## 5 Conclusion

Based on microbiota analysis, no differences were found between SA and CA groups in terms of alpha and beta diversity. Consistent with previous studies, rectal swab samples from SA showed higher relative abundances of *Bacteroides* and *Roseburia*, whereas tissue samples from CA were enriched with potential pathobionts. In addition, *Eubacterium* and *Clostridioides* were increased in both SA and CA rectal swab samples compared with controls. Microbiota profiling of appendiceal tissue and rectal swab samples may provide complementary information for understanding microbiota-associated patterns in appendicitis; however, the diagnostic or predictive utility of these microbial profiles requires validation in larger, independent cohorts using dedicated predictive modeling and diagnostic performance analyses.

## Data Availability

The datasets presented in this study can be found in online repositories. The names of the repository/repositories and accession number(s) can be found in the article/supplementary material.
